# “It has to be better, otherwise we will get stuck.” A Review of Novel Directions for Mental Health Reform and Introducing Pilot Work in the Netherlands

**DOI:** 10.2174/0117450179271206231114064736

**Published:** 2023-11-17

**Authors:** Jim van Os, Floortje Scheepers, Michael Milo, Gijs Ockeloen, Sinan Guloksuz, Philippe Delespaul

**Affiliations:** 1 Department of Psychiatry, University Medical Centre Utrecht, PO Box 85500, 3508 GA Utrecht, The Netherlands; 2 Department of Psychosis Studies, Institute of Psychiatry, Psychology & Neuroscience, King’s College London, London, UK; 3 Milo Health Care Connector and Change Management Consultant, Berlagehof 14, 1067 NB Amsterdam, The Netherlands; 4 Reframing Studio Design Introspector, Bilderdijkkade 50 A11053 VN Amsterdam, The Netherlands; 5 Department of Psychiatry and Neuropsychology, Maastricht University, 6200 MD Maastricht, The Netherlands; 6 Department of Psychiatry, Yale University School of Medicine, New Haven, CT, USA; 7 Mondriaan Mental Health Trust, 6401 CX Heerlen, The Netherlands

**Keywords:** Public mental health, Mental health services, Social care, Recovery college, Mental health, Mental health reform, Social trials

## Abstract

**Background:**

The current state of mental health care in the Netherlands faces challenges such as fragmentation, inequality, inaccessibility, and a narrow specialist focus on individual diagnosis and symptom reduction.

**Methods:**

A review suggests that in order to address these challenges, an integrated public health approach to mental health care that encompasses the broader social, cultural, and existential context of mental distress is required.

**Results:**

A Mental Health Ecosystem social trial seeks to pilot such an approach in the Netherlands, focusing on empowering patients and promoting collaboration among various healthcare providers, social care organizations, and peer-support community organizations, working together in a regional ecosystem of care and committed to a set of shared values. In the ecosystem, mental health problems are examined through the prism of mental variation in context whilst scaling up the capacity of group-based treatment and introducing a flexible and modular approach of (2^nd^ order) treatment by specialists across the ecosystem. The approach is to empower naturally available resources in the community beyond professionally run care facilities. Digital platforms such as psychosenet.nl and proud2bme.nl, which complement traditional mental health care services and enhance public mental health, will be expanded. The capacity of recovery colleges will be increased, forming a national network covering the entire country. GEM will be evaluated using a population-based approach, encompassing a broad range of small-area indicators related to mental health care consumption, social predictors, and clinical outcomes. The success of GEM relies heavily on bottom-up development backed by stakeholder involvement, including insurers and policy-making institutions, and cocreation.

**Conclusion:**

By embracing a social trial and leveraging digital platforms, the Dutch mental health care system can overcome challenges and provide more equitable, accessible, and high-quality care to individuals.

## 1 INTRODUCTION

Around thirty years ago, a comparison of mental health services in European countries concluded that all countries were struggling to develop a sustainable and effective mental health care system [1]. The question arises to what degree this has since changed for the better. Over the past three decades, academic psychiatrists and psychologists have consistently identified “new” disorders and devised corresponding treatments; however, the development of sustainable mental health care systems has not been a central focus and continues to be inadequately explored [2-4].

The Netherlands similarly faces substantial challenges in the provision of mental health care services. A recent government report on health care in the Netherlands states: *It has to be better, it can be better. Otherwise, we will get stuck* [5]. Long waiting lists, an alarming rise in the prevalence of mental disorders in young people, inadequate access to care for those with the most needs, unsafe environments for the mentally ill, dependence on an individual and medicalized approach with rising levels of psychotropic medication use and specialist-administered psychotherapeutic interventions that are scarce and unequally distributed, are among the most pressing issues [6-14]. The current specialist-based system, characterized by a strong emphasis on diagnosis and individual treatment for medical high-risk states and illness in clinical settings, based on prescriptive guidelines derived from symptom reduction group comparisons, is struggling to meet the increasing demand for mental health services. In addition, clinical practice guidelines are rarely updated and rely on controlled data while minimizing the evidence for real-world data as well as issues to do with access and healthcare costs. Recent analyses, echoing earlier calls [6, 10, 11, 15-17], advocate for a transformative approach [18] that tailors affordable and accessible healthcare responses to the unique circumstances of the person [19] while circumventing the medicalization of social and existential needs [20, 21]. But what is the best way to achieve this?

### 1.1 The Complexity of Mental Health Care Change

**Fig. (1):**
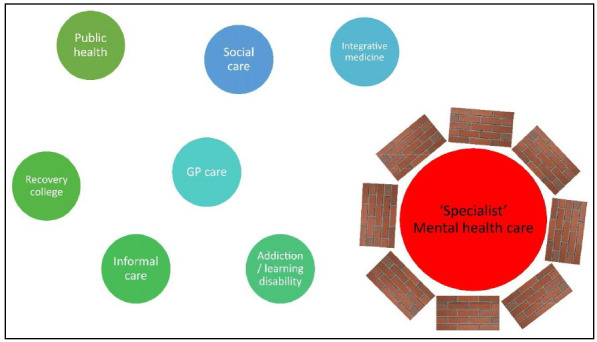
Current state of fragmented care. The mental health care system in the Netherlands can be challenging for individuals to navigate due to its regulated market health care system that creates bureaucratic complexity and separates social care from health care. Additionally, the mental health care system's focus on DSM-diagnoses and evidence-based symptom reduction may lead to a narrow approach to mental health care, which overlooks the broader social and contextual factors influencing individuals' well-being. Fragmentation of services and selective patient selection by providers further exacerbate the issue. The municipal public health service (GGD) is responsible for promoting and protecting public health at a local level, with the OGGZ task focusing on identifying and supporting vulnerable individuals with complex psychosocial or psychiatric needs who may not seek help independently. eCommunities like psychosenet.nl and proud2bme.nl offer accessible online support and digital interventions for individuals experiencing psychosis or eating disorders, respectively, breaking down stigma and isolation often associated with mental health issues and demonstrating the potential of digital platforms to complement traditional mental health care services and enhance public mental health. The Netherlands' social care system supports vulnerable individuals and promotes well-being through a range of services, with responsibility largely lying with municipalities to ensure tailored, community-based approaches. The lack of integrated collaboration between mental health care and social care is a pressing problem, given the fact that mental health care consumption in the Netherlands is strongly driven by socioeconomic factors [26, 27]. Recovery colleges focus on empowering individuals to develop self-management skills, build resilience, and foster personal growth, providing a safe, inclusive, and supportive environment. The GP serves as the primary healthcare provider and gatekeeper for specialist services, including mental health care, with the POH-GGZ providing support in assessing, treating, and referring patients with mental health concerns. Addiction services offer outpatient, inpatient, and community-based treatments while learning disability services offer assessments, personalized support, therapy, and guidance for individuals with learning disabilities and their families. The complementary and alternative medicine sector operates alongside conventional healthcare, offering a range of therapies and treatments outside of mainstream practices. Informal mental health care in the Netherlands encompasses a diverse range of non-professional support systems, including religious institutions, voluntary organizations, resource groups, family members or relatives, and community centers, that play a crucial role in promoting well-being and assisting individuals with mental health needs.

The complexity of change in mental health care systems in industrialized countries has long been recognized, as in the Netherlands [22, 23]. In this country, two significant reforms enacted since the mid-2000s stand out. The 2006 reform abolished the distinction between public and private insurance, establishing a single universal social health insurance system and incorporating competition that allows citizens to select between different insurance plans - that offer minimal differences because the basic plan is very comprehensive and imposed by the government. The impetus for quality improvement within the healthcare system stems from regulated competition, wherein insurance companies, rather than individuals, select healthcare providers. These providers predominantly emphasize cost efficiency and frequently engage in case selection, often prioritizing less complex cases. In addition, a recently introduced long-term care reform aims to shift from publicly provided care to increased self-reliance for citizens and an expanded role for municipalities in organizing care. A specific area of concern is the integration and collaboration of new governance structures and responsibilities within long-term care. The regulated market system of healthcare in the Netherlands has led to some perverse incentives for economically driven selection of patients with less severe problems and avoidance of more complex patients [11]. This system may inadvertently contribute to a widening gap in access to appropriate care for those who need it most [24]. As mental ill-health comorbidity is typical for increased instability, severely ill patients are increasingly at risk. The regulated market system has also resulted in an increasingly fragmented mental health care landscape (Fig. **1**). In addition, the separation and lack of collaboration between social care and mental health care in the Netherlands has created additional distinct challenges in addressing the mental health needs of the population [25].

Another critical issue in the Dutch mental health care system is the observation of rising levels of mental distress among young people, which are widely thought to be attributable to existential and social factors that may not be best addressed within the individualized specialist-oriented mental health care system [28]. The increasing prevalence of mental distress among young people thus underscores the need for a more comprehensive and integrated approach that addresses the root causes of distress and promotes resilience [29]. However, the lack of a well-developed public health perspective in the Netherlands has impeded efforts to address mental health challenges at the population level [22]. A more robust public health approach, incorporating mental health promotion and prevention strategies, as well as non-medical early intervention and digital strategies, could help to identify and address the underlying factors contributing to mental health difficulties, particularly among vulnerable populations [30].

### 1.2 The Case for Transition towards a Collaborative Public Mental Health System

Here, we argue that a paradigm shift is required, taking into account the complexity of change in health care in industrialized countries, transitioning from a medical-specialist-based approach to a system that is co-grounded in the principles of public mental health, with which the medical-specialist system seeks flexible and modular collaboration in an ‘ecosystem’ of mental health [8, 31]. Public mental health is a population-based approach that emphasizes prevention through risk reduction, non-medicalized early intervention, reduction of stigma and disparity, and salutogenesis or the promotion of resilience and mental well-being [32-35]. It seeks to address the social determinants of mental health, reduce disparities in access to care, de-medicalize the population's mental health narrative, promote scientific evidence of the power of (group-based) relational rituals in restoring mental health, foster collaboration across sectors, and create on-the-ground and online peer-supported environments for people to thrive. It seeks to implement a core collectivistic approach towards mental health that is supported by a more modular and flexible individual, specialist approach that often is delivered through a 2^nd^ order task-shifting approach rather than a first-order treatment approach.

This article describes a social trial introducing such a transformation in various regions of the Dutch mental health care system and offers recommendations for implementing this change based on the early pilot activities. We will present which components of transformation are currently required in Dutch mental health care, how a social trial tackling these specific challenges was set up, how a trajectory of change was set up to facilitate each component of transformation, and how the transformation will be monitored and evaluated.

### 1.3 Which Components of Transformation are Required and Why?

In order to transition from a fragmented, overly specialized medical system to a more public mental health care-focused approach, several critical domains must be addressed. These are listed in Table **1** and will be discussed in more detail below.

### 1.4 From an Individual to a Collectivistic Emphasis

In the current mental health system, mental problems are primarily perceived as the decontextualized manifestation of an illness ‘in the head’ that requires specialist diagnosis and treatment. However, the yearly prevalence of mental disorders in the Netherlands has risen from 1 in 5 in the period 2007-2009 to 1 in 4 currently [36]. This increase indicates, first, that – likely modifiable – environmental factors are massively impacting mental health and may be mitigated by a public mental health risk reduction approach. There is solid evidence that poverty, deprivation, and a range of well-established environmental factors drive mental distress [37, 38] that can be targeted in the context of public health interventions [39-41]. Second, the increase in prevalence means that any mental health system based on individual specialist treatment will, per definition, be massively overtasked. These developments, therefore, point toward an urgent need for a more collectivistic, population-based approach to thinking about mental health. Given the particularly strong increase in mental distress in young people, there are increasingly loud calls for public mental health strategies [42], with some countries moving towards a public mental health focus for (youth) mental health [43-45].

### 1.5 From Power Imbalance to Cocreation

The current mental health care system often suffers from power imbalances between mental health professionals and patients [46]. Mental health professionals typically have the power to diagnose and label patients with mental health conditions. This can be unhelpful and stigmatizing and can reinforce the power dynamic between the professional and the patient [47]. Mental health professionals are seen as having specialized knowledge and expertise, which can lead to patients feeling like they have to defer to the professional's judgment rather than being able to fully participate in decision-making. In addition, mental health professionals often have institutional power and resources within mental health systems and organizations, which can reinforce the power imbalance between the professional and the patient and limit the ability of patients to advocate for themselves [48]. This power imbalance can be redressed by (i) focussing on the daily life adaptational strategies that are the core of recovery and for which citizens and their involved network are the prime references for information; (ii) systematic patient education, for example, from the perspective of lived experience; (iii) using personalized metrics of, for example, personal goals and directions of change; and (iv) the introduction of public health terminology and evidence into policy and practice, such as accepting that recovery is a subjective experience that the individual should determine; that recovery from mental health issues is possible for many individuals; that formal diagnosis is not a reliable basis for treatment; that treatment is one of many paths to recovery; and the effects of mental health issues are varied and complex [49].

### 1.6 From Traditional Specialist Knowledge and Settings towards Equity between Experiential and Specialist Knowledge and Settings

A narrow evidence-based model of mental health care, focused on symptom reduction and functioning, carries the risk of not meeting social and existential care needs [9] [50]. Recovery colleges focus on providing education and support to individuals with mental health issues, with the goal of overcoming social defeat and internalized stigma, promoting recovery and personal growth. They are based on the principles of recovery-oriented practice, which prioritize the needs and experiences of individuals with mental health issues and aim to support their journey toward recovery [51, 52]. Individuals can enroll in courses and workshops that cover a wide range of topics related to mental health and well-being, designed and delivered by people with lived experience who bring their own expertise and experience to the teaching and learning process. Recovery colleges typically emphasize peer support, collaboration, and shared learning, with the goal of empowering individuals to take an active role in managing their own mental health and well-being. By promoting the recovery-oriented practice and involving mental health service users in the teaching and learning process, recovery colleges can help to promote recovery and improve mental health outcomes for individuals with mental health issues. Evidence suggests that recovery colleges are popular with student users and that college attendance helps with the process of recovery [53, 54]. Importantly, colleges can engage people who do not wish to interact with services and bring about self-reported improvements in several areas, such as self-confidence, self-esteem, and self-understanding [55]. Recovery colleges are increasingly popular and continuously adapting to the needs of users but remain structurally underfunded in high-income countries [56, 57]. Innovative ways to introduce more recovery-oriented structures alongside existing systems are required [58].

### 1.7 From Treating High-risk States/illnesses to Reducing (vulnerable) Population Illness Rate

Treating illness is not an effective way to improve health at the population level [59]. Similarly, a medical high-risk strategy of prevention, focusing on identifying and intervening with individuals who are considered to be at a higher risk of developing mental health disorders based on specific criteria, such as genetic predisposition or subclinical symptoms, is not effective at the population level because of the ‘prevention paradox’ [60]. Indeed, it may have unwanted iatrogenic effects, inducing higher levels of mental distress [61]. A population-based risk-reduction strategy of prevention in mental health care focuses on addressing the underlying determinants of mental health and implementing interventions that target the entire population or specific vulnerable subpopulations [35]. Population-based strategies often emphasize the importance of addressing important social determinants of mental health, such as socioeconomic status, education, loneliness, lack of connectedness, trauma, social defeat, employment, housing, and social support. These factors have a significant impact on mental health outcomes and can be addressed more effectively through population-level interventions, as opposed to medical strategies that primarily focus on individual risk factors. Population-based strategies thus can help prevent mental health issues from arising in the first place, thereby reducing the overall burden on mental health care systems. In contrast, medical high-risk strategies often involve intervening after symptoms have already emerged or when individuals are already at an elevated risk, which typically is less effective in reducing the overall prevalence of mental health disorders. Population-based strategies often promote protective factors and resilience-building, which can benefit a wide range of individuals, even those not considered to be at high risk. Medical high-risk interventions also bring with them a significant risk of medicalization, creating dependence and stigma. They are also relatively ineffective as the ability to predict outcomes in people with mental complaints is low, and there is little evidence that interventions can reduce ‘transitions’ from ‘risk state’ to ‘illness state’ [62, 63].

### 1.8 From ‘Disorderism’ to a Contextual Understanding of Mental Distress

It has been cogently argued that there may be several important clinical, conceptual, and relational advantages in moving away from the current practice of a narrow diagnosis of mental illness as a biological problem in the brain [64-66]. Currently, the importance attached to categorical diagnosis in the mental health system can be seen as a systematic attempt to decontextualize a person’s experience, a practice that has been referred to as ‘disorderism’ [67], paving the way to interventions that can ‘fix’ the brain. An alternative approach that shows promise [68] is to reframe the language used to describe mental distress, shifting towards a contextual understanding that recognizes consciousness and meaning as key tools for navigating complex environments. This perspective acknowledges the dynamic interplay between bottom-up sensory information and top-down predictions informed by previous experience, highlighting the importance of individualized assessments that consider the unique contexts and circumstances of each person [69]. The contextual public mental health model recognizes that mental health is influenced by a wide range of factors, including biological, psychological, social, existential, and cultural factors [37, 38] and the process of meaning-making that emerges from these factors. This holistic understanding allows for a more comprehensive approach to mental health, which takes into account the complexity and interconnectedness of different aspects of a person's life. There is also more room for recognizing that each person's experiences and circumstances are unique and, therefore, require a more personalized and interactive approach to mental health care. This allows for more individualized treatment plans that take into account a person's unique needs and circumstances. As the contextual model of mental health emphasizes the importance of self-awareness and self-regulation, it can empower individuals to take an active role in their own mental health and well-being. This can lead to greater agency and a sense of control over one's life. It also calls for a more socially connected approach, as it promotes the importance of social connections and support systems for mental health and well-being. It may result in a more community-oriented approach to mental health care, which focuses on building and strengthening social networks and support systems. Finally, a contextual model of mental health is built on the notion of the importance of diversity and cultural factors in mental health and well-being. This allows for a more inclusive approach to mental health care, taking into account the unique experiences and perspectives of different individuals and communities.

### 1.9 From Specialist Interventions towards Risk Reduction, Health Promotion, Health Literacy, Stigma Reduction, Building Resilience, Protective Factors, and Non-medical Early Intervention

Evidence suggests that specialist interventions work to a large degree through non-specific mechanisms that may be offered more effectively in groups in a community context [9]. Examples of these are:

o Health promotion interventions that aim to improve mental health and well-being by promoting healthy behaviors and lifestyles. Examples of health promotion interventions include encouraging physical exercise, promoting healthy eating habits, and promoting adequate sleep.

o Resilience-building interventions help individuals develop the ability to cope with stress and adversity and to bounce back from challenging life events. Examples of resilience-building interventions include mindfulness training and stress management techniques.

o Mental health literacy interventions are aimed at improving knowledge and awareness of mental health issues and promoting positive, demedicalized attitudes towards mental health. Examples of mental health literacy interventions include mental health education programs, social media campaigns, and public information campaigns. It also includes mental health education for young people, such as early symptoms to look out for, resources to access education, and resources for how to seek help.

o Stigma reduction interventions can reduce negative attitudes and beliefs about mental health issues and promote understanding and acceptance of individuals with mental health issues. Examples of stigma reduction interventions include anti-stigma campaigns, education programs, and media campaigns.

o Protective factor promotion interventions are focussed on promoting the development of protective factors, such as social support, positive relationships, and healthy coping mechanisms, which can help individuals manage stress and other life challenges and reduce the risk of mental health issues. Examples of protective factor promotion interventions include social skills training, relationship-building interventions, and stress management techniques.

o Risk reduction: Risk reduction interventions aim to reduce the risk of mental health issues by addressing factors that increase the likelihood of developing mental health issues. Examples of risk reduction interventions include substance abuse prevention programs, early intervention for trauma, and suicide prevention programs. Other factors that can be included are loneliness, poverty, family problems, housing problems, and unemployment.

o Non-medical early intervention: non-medical early intervention programs aim to provide support and intervention for individuals experiencing mental health issues before the need for more intensive medical interventions. Examples of non-medical early intervention programs include ‘@ease’ centers for young people, peer support programs, crisis hotlines, and community-based support services.

o Interventions that focus on building an inclusive and supportive society with cohesion and social safety as core values. Examples are programs that promote diversity, social support, positive group experiences, and meeting places where people can exchange their experiences, expertise, and support.

### 1.10 From Limited Specialist Capacity to Unlimited Capacity through Task-shifting, Simplifying Interventions, Group-approaches and Ecommunities

Task-shifting is a concept in public mental health that involves delegating specific tasks or responsibilities to non-specialist healthcare workers or community-based workers. This is often done in situations where there is a shortage of trained mental health professionals or where demand for services exceeds the capacity of the system. Task-shifting has been used effectively in low- and middle-income countries to improve access to mental health care in underserved areas [70] and is one of the ways in which high-income countries can learn from strategies developed in low- and middle-income countries [71]. Task-shifting in high-income countries is urgently required, given the phenomenon of the specialist care paradox. Here, the majority of specialized mental health practitioners primarily address well-defined disorders, as classified by the DSM-5R, while frequently neglecting the intricate, co-morbid cases. This paradoxical situation results in specialists attending to less complex, average cases rather than devoting their expertise to the highly complex, co-morbid presentations that demand their skills the most. To optimize the allocation of resources and expertise, it is essential to recalibrate this approach, thereby enabling specialists to focus on the most challenging cases.

In high-income countries, rising levels of mental ill-health among young people have led to increased demand for mental health interventions. However, there is often a shortage of trained mental health professionals, such as psychiatrists and clinical psychologists, to meet this demand. Task-shifting can be used in this context to increase the capacity of the mental health system by delegating specific tasks to nurse specialists, non-specialist healthcare workers, or community-based workers.

For example, in the context of delivering therapy to young people with mental health problems, task-shifting could involve delegating – but also providing supervision – certain aspects of therapy, such as psychoeducation, skills training, psychotherapies, and medication use to non-specialist healthcare workers or community-based workers. This could free up mental health professionals to focus on more complex cases while still ensuring that young people receive appropriate care and support. Working in a group can be particularly effective in the context of task-shifting, as non-specialist healthcare workers or community-based workers can be trained to deliver group therapy sessions under the supervision of a mental health professional.

Another important, related issue has to do with the complexity and timing of mental health interventions. The efficacy of various therapeutic approaches has been widely studied, revealing minimal differences in their effectiveness. This raises the question of what components are necessary for a therapy to be successful and whether elements can be removed without compromising therapeutic outcomes. Identifying these minimal interventions is crucial for developing cost-effective, easily accessible treatments that require little to no input from highly trained mental health professionals [72]. Research suggests that offering timely access is crucial [73].

Group approaches to delivering therapies in mental health services can also be used to greatly increase the capacity of the system. There is solid evidence that psychotherapy delivered in groups is as effective as individual psychotherapy for the majority of people seeking help [71]. Indeed, group therapy has many advantages. Group therapy provides clients with an opportunity to learn from and receive support from others who are undergoing similar experiences and challenges. This can help reduce feelings of isolation and foster a sense of connection and understanding. In a group therapy setting, there is a lower risk of developing a dependency on a (dominant) therapist, as attention is distributed among multiple clients. This promotes more equitable and healthy therapeutic relationships. Group therapy enables clients to establish therapeutic relationships with one another, facilitating communication, providing and receiving feedback, and collaboratively working towards common goals. This offers valuable skills for building and maintaining healthy relationships outside the therapeutic environment. Finally, group therapy inherently offers a rich tapestry of experiential knowledge and access to a recovery-based learning model, complementing therapy focused on symptom reduction.

Research on emerging internet-based practices reveals the potential of translating many elements of recovery colleges to digital platforms. Popular eCommunities in the Netherlands, such as psychosenet.nl and proud2bme.nl, drawing millions of yearly visitors, are exemplary in this regard. They incorporate essential aspects of mental health recovery colleges, such as education, peer support, social engagement, demoralization, self-management, and self-expression [74, 75]. Their interactive elements encompass features like (group)chat, forums, and online consulting. Beyond these foundational elements, eCommunities also present a novel avenue for the introduction and promotion of various free eHealth and mHealth interventions. Users can explore and experiment with these digital solutions at their convenience. One of the distinguishing benefits of disseminating such digital interventions within the context of eCommunities is the innate support system it provides. Community members can assist one another in navigating these digital tools, sharing experiences, offering guidance on their usage, and advising on the potential effectiveness of different interventions for varying situations. This peer-guided exploration can foster an element of ‘blended’ eHealth and collective learning. By consolidating these features into an accessible and inclusive online platform, eCommunities stand at the forefront of a public mental health approach that transcends geographical and physical limitations, ensuring a more democratized access to mental health resources and support.

eHealth and mHealth solutions frequently emulate traditional office-based interventions. However, while these conventional approaches aim to provide insights and skills intended for application in everyday life, they often do not result in a process of “generalization” that is effective. For the first time in the history of psychosocial interventions, assessments can now be conducted within the context of individuals' daily lives, and skill development can be directly integrated into situations where help-seekers experience difficulties. This not only facilitates learning but also enables the creation of therapeutic aids that do not necessitate a therapist's presence, empowering individuals to function autonomously and resiliently through context-sensitive cues and support.

### 1.11 From an Economically Driven Fragmented approach to Care to a Values-based Coordinated Approach to Care

In countries like the Netherlands, the mental health care system has become production- and income-driven rather than values-driven [76, 77]. This means that the focus has shifted from providing high-quality care and promoting the well-being of patients to maximizing revenue and minimizing costs. This shift has been manifested in an increased emphasis on efficiency and productivity, reduced focus on patient-centered care treatment with a focus on standardized and impersonal treatment plans, with little consideration for the patient's individual needs, overreliance on medication as the ‘quick and easy’ solution to mental health issues; reduced emphasis on prevention and non-medical early intervention as in a production-driven system, resources may be allocated primarily to crisis interventions and emergency services; and neglect of social and environmental factors. A production- and income-driven mental health care system, in combination with a focus on biological factors and pharmacological interventions, can undermine the values that are essential to promoting the well-being of individuals with mental health issues [2]. To create a more values-driven system, mental health care providers must prioritize patient-centered care, prevention and early intervention, and holistic approaches to mental health that take into account the social, economic, and environmental factors that shape individuals' experiences.

Shared values such as humanity, relationism, authenticity, collaboration, cocreation, equity, inclusivity, and shared responsibility can play a crucial role in creating a coordinated ecosystem of care in a mental health system that is fit for the moral era of medicine [78]. Importantly, the value of *collaboration* involves working together with others to achieve a common goal. In the mental health system, collaboration between different providers, organizations, and stakeholders can help to bridge gaps in care, improve communication and coordination, and create a more seamless and integrated system of care. In a field where multidisciplinary approaches are of paramount importance, providing support and expertise from various resources in parallel is essential for the most critically ill individuals. The engagement of partners may be hindered if it leads to isolation from other stakeholders [79, 80]. The cornerstone of care in an ecosystem design should be a unified, concurrent collaboration rather than a sequential one. *Cocreation* is a process of collaborative and inclusive decision-making that involves bringing together multiple stakeholders, including those with lived experience of mental illness and mental health professionals, to work together on equal footing to create solutions that meet the needs of everyone involved. It involves acknowledging and valuing the unique contributions and expertise of each participant and creating a space for their voices to be heard and their ideas to be considered. When mental health care providers prioritize the *humanity* of their patients, they view them as whole individuals with unique needs, experiences, and goals [81]. This approach can foster trust, empathy, and respect between patients and providers and can lead to more personalized and effective care. A *relationist approach* to mental health care emphasizes the importance of building positive relationships between patients, providers, and other stakeholders in the mental health system. *Authenticity* involves being honest, transparent, and genuine in one's interactions with others. When mental health care providers prioritize authenticity, they can establish a sense of trust and mutual understanding with their patients and create a safe and non-judgmental environment for open communication and exploration of mental health issues. *Equity* involves recognizing and addressing the systemic barriers that prevent individuals with mental health issues from accessing quality care and support, and respect involves valuing the unique experiences, perspectives, and identities of all individuals and treating them with dignity and empathy. *Inclusivity* involves recognizing and embracing diversity in all its forms, including differences in culture, language, ethnicity, race, gender, sexuality, and ability. By prioritizing inclusivity, mental health care providers can create a more welcoming and accessible environment for individuals with mental health issues and promote a culture of acceptance, understanding, and celebration of diversity. Finally, *shared responsibility* involves recognizing and acknowledging the role that different stakeholders play in supporting mental health and well-being and taking collective action to address gaps and barriers in the system. By sharing responsibility for mental health care, stakeholders can work together to identify and address systemic issues and promote a more equitable and inclusive mental health system.

### 1.12 Examples of Public Mental Health Initiatives in Industrialized Countries

There have been several public mental health initiatives in industrialized countries aimed at promoting mental well-being, preventing mental health issues, and providing care and support for those affected, with at least some evidence of effectiveness. Some of these initiatives include Mental Health First Aid (MHFA) in Australia and the USA - this is a program that trains individuals to identify and respond to signs of mental health issues in others [82]; the “Friendship Bench” project is a mental health intervention that originated in Zimbabwe and has since been implemented in other locations, including New York City. The intervention involves the use of community health workers trained in problem-solving therapy, who offer support to individuals experiencing common mental health problems like anxiety and depression. These conversations typically take place on wooden benches located in public spaces, such as parks and community centers [83]. The Icelandic Model of Adolescent Substance Use Prevention is a theoretically grounded, evidence-based approach to community adolescent substance use prevention that has grown out of collaboration between policy makers, behavioral scientists, field-based practitioners, and community residents in Iceland. The intervention focuses on reducing known risk factors for substance use while strengthening a broad range of parental, school, and community protective factors [84]. Time to Change in the UK - A campaign aimed at reducing mental health stigma and discrimination [85]; Improving Access to Psychological Therapies (IAPT) in the UK: A program that provides access to evidence-based psychological therapies for common mental health problems [86] and The Australian Headspace program [86]; and the European Alliance Against Depression (EAAD): A network of organizations focused on combating depression and preventing suicidal behavior through community-based programs [87].

Similarly, integrating aspects of social care, mental health care, and community-based recovery initiatives is an essential public mental health approach for promoting mental well-being and supporting individuals with mental health issues. Some well-known examples of such initiatives in high-income countries are Assertive Community Treatment (ACT) in the USA: ACT is a multidisciplinary, team-based approach that provides comprehensive, integrated community-based support for people with severe mental illnesses [88]; the Recovery College model in the UK - focusing on self-management, empowerment, and social inclusion, integrating care and recovery initiatives [89]; Partners in Recovery (PIR) in Australia: PIR is a coordinated integrative approach that aims to support individuals with severe and persistent mental illness, along with complex needs, fostering collaboration among service providers and promoting personalized care [90]; Housing First in Canada - this is an evidence-based approach to ending homelessness, particularly for individuals with mental health issues. The model prioritizes providing permanent housing to people experiencing homelessness in an attempt to integrate care and recovery initiatives [91]. Other programs that focus on more personal and social aspects of care and recovery are Peer-Supported Open Dialogue [92, 93], Wellness Recovery and Action Plan (WRAP) [94], Individual Placing and Support (IPS) focusing on rehabilitation in work and education [95], and variants of assertive community treatment (ACT) such as resource-group ACT and flexible-ACT [96].

While the examples of public mental health initiatives in industrialized countries highlighted above are noteworthy and demonstrate a genuine commitment to promoting mental well-being and offering support, none of these initiatives were established within the framework of a comprehensive mental health reform strategy that addresses all the varied facets of needed changes. These programs, though potentially effective in their localized objectives, represent disparate efforts to tackle specific elements of the broader mental health challenge. Whether focusing on mental health first aid, stigma reduction, access to therapies, or community-based support, none encompass a holistic approach targeting defragmentation of services, prevention, early intervention, care, risk reduction, group-based approaches, a contextualized model of mental distress, recovery, and societal integration simultaneously. A true comprehensive strategy would require a multifaceted approach that interlinks all these dimensions, ensuring that no aspect of mental health care is overlooked.

### 1.13 How Can Comprehensive Change be Implemented? Introducing the Mental Health Ecosystem Social Trial in the Netherlands

The complexity of mental health care and the lack of a comprehensive overview make it difficult to identify how the system should or could change. The best approach towards change, therefore, is to conduct action-oriented participatory research in several regional areas, focused on transformation, supported by a national learning community to facilitate further implementation and building on previous public mental health initiatives in other countries with comparable mental health care systems. In 2022, a think tank consisting of representatives from health insurers, patient organizations, the associations of municipalities, mental health care providers, and academia held a series of meetings that resulted in the main principles for this type of action research, supported by the Netherlands Organization for Health Research and Development. A project call was organized, grounded in change management as well as scientific and recovery principles, aimed to help transform the mental health sector and its regional partners (social care, recovery academies, general practitioners, public health services, integrative medicine, informal care) towards an improved *Ecosystem of Mental Health*, after the Dutch term *Ecosysteem Mentale Gezondheid*, abbreviated as ‘GEM’ [97]. Regions interested in setting up GEM were eligible to apply for funding. This resulted in social trial action research in five regions in the Netherlands that are currently ongoing. The transformation process in these social trials involves 7 “change workshops” and includes impact assessment studies.

Given the earlier described components of transformation towards a collaborative public mental health system, GEM addresses the following specific key challenges and solutions in the Dutch health care setting:

1. *Challenge 1*: There is fragmentation of services between mental health services, social care, recovery academies, integrative medicine, informal care, GP care, addiction services, learning disability services, and youth mental health care. *Approach*: GEM brings together workers from social care, mental health services, recovery academies, integrative medicine, and other professionals (without merging separate organizations) in a values-driven, demedicalizing local ecosystem. For the pilots, the scale is small (10-15.000 population) and local to facilitate collaboration. The policy and values scale, however, is at the level of the entire municipality (100.000-200.000). Key values include relationships, humanity, equality, context-focused collaboration, and peer support.

2. *Challenge 2*: Clients have limited choice in mental health services and are assigned treatment based on a (centralized triage towards ‘specialist’ diagnosis and evidence-based guidelines that do not focus enough on social participation and existential recovery and, therefore, insufficiently address needs. *GEM approach*: Individuals can choose where in the ecosystem they want to begin addressing their issues, whether at a recovery academy, a social care group, a mental health group, or an eCommunity, and choose between the elements in the ecosystem. Professionals, on the other hand, are trained to assess problems in a holistic way and to propose solutions from a broader perspective, for example, using the Network-intake https://sociaalweb.nl/nieuws/netwerkpsychiatrie-hoe-dan/).

3. *Challenge 3*: The Dutch healthcare system is a mix of private and public components, where basic health insurance is required for all residents, and private insurers offer these plans with government-regulated coverage and pricing. However, the current system has resulted in high costs, complexity for consumers, inequality, administrative burden for mental healthcare providers, and missed opportunities for prevention. *Approach*: For GEM to succeed, limited changes are necessary for healthcare purchasing and reimbursement. Active collaboration within the local ecosystem of mental health should be conditional for health care purchasing, and flexible reimbursement rules are needed to allow for a more consultative approach of specialists within the ecosystem.

4. *Challenge 4*: Psychological distress is unnecessarily funneled towards a medical solution due to the increasing tendency to view it through the lens of specialist diagnosis, even though it often has social or existential causes that require different responses. *GEM approach*: In the mental health ecosystem, psychological distress is always considered within its context, with a focus on social and existential factors, as described earlier [68]. This prevents unnecessary medicalization of social and existential issues, which receive a diversified response in the ecosystem [98]. Systematic collection of key exposomic information can make care more contextual and avoid unnecessary medicalization.

5. *Challenge 5*: The yearly prevalence of diagnosable mental disorders in the Netherlands is 25% [36], but the Dutch mental health system only has the capacity to serve 9% of those diagnosed yearly. Consequently, the system is permanently overburdened, and people with severe problems face difficulties accessing care. *Approach*: GEM significantly increases the capacity for social, medical-psychological, and existential treatments in a cost-neutral manner by (i) Providing more than 80% of all treatments in groups, (ii) expanding the availability of dynamic online eCommunities (online self-management/recovery centers) like www.psychosenet.nl [74, 75], and (iii) developing tools to foster autonomous development of resilience with peers and involved members of the environment.

6. *Challenge 6*: There is a strong need for increased social holding and participation for people with non-linear behavior who struggle to maintain stability in society. *GEM approach*: Enhanced collaboration between social care, recovery academies, and mental health services, creating space for peer-supported open dialogue, retreats, and resource groups for social holding, individual placement and support (IPS), social enterprising and entrepreneurship, and social care “well-being on prescription” for participation.

7. *Challenge 7*: There is a high demand for recovery academies where people can attend learning groups to shape their lives despite mental health issues. *GEM approach*: the input of experiential expertise is increased and integrated into a values-driven ecosystem of collaboration with social care and mental health services. Access is not limited to ‘ill’ persons but forms an integral part of the local ecosystem.

8. *Challenge 8*: Mental health services have become isolated in specialist care pathways, focusing on long-term specialist treatment rather than clients' real-life needs. *GEM approach*: Mental health services will shift from isolated specialist care pathways to a flexible, modular, and improvisational approach on demand. Specialists focus on treatment that supports clients' efforts to regain control of their lives, often through second-order treatment involving others in the ecosystem.

9. *Challenge* 9: There is a need for public mental health in the form of programs aimed at risk reduction, promotion of resilience, and mental health literacy in young people, including recognizing mental distress and how to initiate self-management approaches such as the formation of a resource group and finding one’s way to eCommunities and other public programs. *GEM approach*: as most of the public mental health work in terms of risk reduction, promoting resilience, and mental health literacy remains to be developed in the Netherlands, it cannot be expected that GEM, being a social trial at specific locations, will offer a national public mental health resource from the start. However, while the GEM initiative recognizes its limitations in directly crafting a national public mental health strategy due to its localized nature, it is determined to pave the way for broader applications. GEM is committed to establishing a solid foundation that can underpin a more expansive public mental health strategy. By actively engaging with health policymakers, GEM seeks to ensure that its findings and recommendations are integrated into the broader health care framework. This would be a significant step towards achieving a harmonized approach, where both individual-level mental health care and public mental health interventions coexist seamlessly.

A graphic summary of the GEM approach is provided in Fig. (**2**).

The figure depicts the “changed” situation evolving from the fragmented state in Fig. (**1**). Components of the ecosystem have committed, on the basis of shared values, to collaboration in a public mental health system. Components commit to shared values in the ecosystem, based on the cocreation of care with stakeholders and social holding for vulnerable individuals. At the same time, important qualitative changes are introduced that are each implemented through a series of change workshops in which new practices are cocreated based on the principles in the text boxes: flexible choice on where to start in the ecosystem, a focus on group-based approaches in mental health care and a focus on flexible consultation and 2^nd^ order treatment in the ecosystem; a contextual view of mental distress as starting point; a network of recovery colleges covering the country; a public health resource of eCommunties; integration of integrative medicine approaches; and integrated collaboration with social care, addiction care and learning disability care.

### 1.14 GEM Change Strategy

The GEM change strategy is informed by previous experience of mental health reform as well as academic theory with regard to implementing change [1, 52, 99-105]. GEM draws from the concept of transition management, an approach to dealing with complex societal problems and fostering sustainable development. According to this model, achieving transformation requires long-term strategic thinking, integrated policies, and a more participatory, multi-actor approach [105]. GEM thus works with various components of transition management and presents it as a potential framework for guiding local mental health care transformation.

The GEM regions start with a series of commitment meetings involving all intended partners within the regional ecosystem. These meetings foster discussions centered on the ecosystem's core values and the commitment to work in accordance with them. This lays the foundation for a 'Binding Journey Program,' which includes the design of Change Workshops for each of the 8 challenges discussed earlier. The workshops facilitate collaboration between individuals with lived experience, volunteers, and professionals from the entire spectrum of healthcare and welfare. Change Workshops are integral to the first phase of the transition process in GEM Regions.

Drawing upon theory and best practice in transitions and system innovations [105], the Change Workshop program is structured into eight steps. Challenges (1) and (2) target leaders (*e.g*., managers, team leaders, directors) of the local coalition, and challenge (3) involves local and national parties, including insurers and several health care authorities. Subsequent 'Design Workshops' (steps 4 to 8) consist of five iterative phases executed over multiple sessions:

o Engage in dialogue about the underlying values of the Change Workshop as a network team, fostering “conscious incompetence” and delineating the shared learning experience based on this insight;

o As a team, systematically extract local (or national) best practices, examples, or instruments that align with the identified values to develop the first version of an experiment;

o Participants form an implementation team and initiate the first experiment;

o Accumulate experience through the experiment and suggest ways to integrate it into the ecosystem;

o Participants collate experiences and devise a plan for the subsequent phase, encompassing organizational preconditions and success indicators.

Each workshop is spearheaded by a local owner, a national 'expert,' and the GEM team serving as facilitators.

**Fig. (3):**
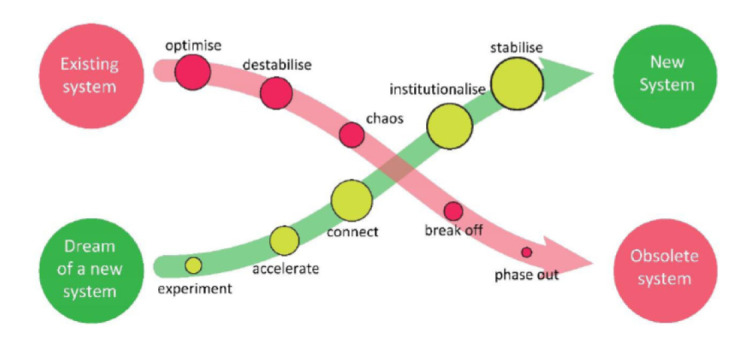
Change strategy in regions of GEM social trial.

Upon completing the Change Workshop cycle, each workshop generates a proposal. Collectively, these proposals outline the prerequisites for the first iteration of the local Mental Health Ecosystem. The ecosystem's “decision-makers” are then tasked with facilitating this transition, recognizing that it is not a project operating alongside the existing system or an improvement of the current state (“better collaboration”). Instead, it represents the first step towards a new system, necessitating shifts and the eventual phasing out of the old system [105] (Fig. **3**).

### 1.15 GEM Research and Monitoring Instrument

The evaluation of mental health services has traditionally focused on individual symptom reduction outcomes, such as symptom severity, improvement rates, and remission. Although these measures provide valuable insights into the effectiveness of interventions, they may not adequately capture the overall quality of mental health care services. In this section, we argue that a population-based approach, which analyzes patterns of small-area health care use and other relevant parameters, offers a more comprehensive understanding of the quality of mental health services.

A population-based approach encompasses a broader range of indicators related to mental health care consumption, social predictors, and clinical outcomes and is aligned with sustainable development goals [106]. These indicators include:

o Patterns of small area health care use: This involves examining compulsory admissions, hospitalization rates, readmission rates, 'revolving door' admissions, dropping out of care, continuity of care, medication use, psychotherapy use, somatic health care use, and social care use.

o Social predictors: Analyzing the relationship between mental health care consumption and small-area levels of socioeconomic deprivation, as well as demographic predictors and health indicators, including medical leave of absence and school absenteeism, allows us to assess whether the level of mental health care expenditure is appropriate for the population in question.

o Coherence with social care and youth mental health care: Evaluating the degree to which small area mental health care use is coherent with social care and youth mental health care helps ensure that resources are effectively allocated and integrated.

o Diagnostic group analysis: Investigating small area parameters for different (trans)diagnostic groups, particularly severe mental illness, addiction, and common mental disorders, helps determine whether there is a good balance in expenditure and no groups are excluded.

o Clinical outcomes: Examining small area clinical outcomes, such as the number of suicides, level of self-harm, population psychotropic medication use, level of diabetes and other somatic complications of chronic psychotropic medication use, employment rate of psychiatric patients, recovery college attendance, rate of homelessness, and incarceration of psychiatric patients, provides a more holistic view of the quality of mental health services.

**Fig. (4):**
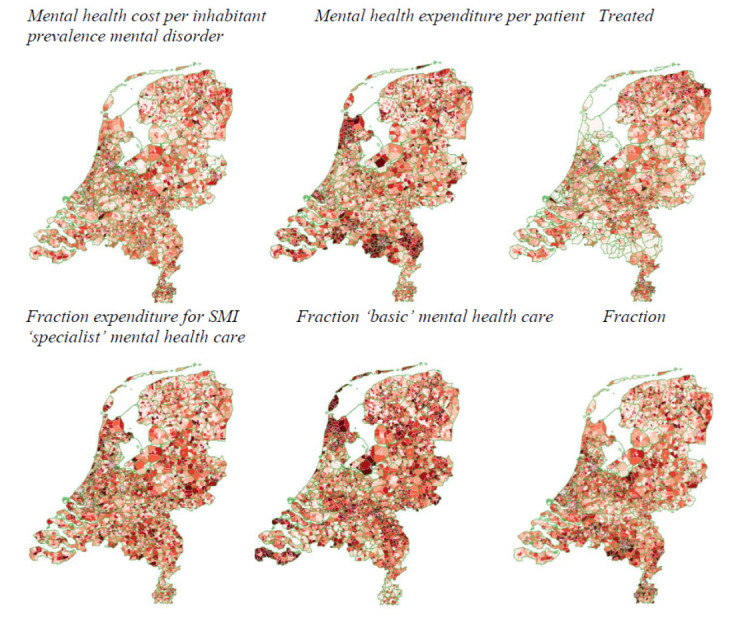
Benchmarking mental health service indicators at the small-area level (4-digit postal code; municipal boundaries in green) using national data 2015-2020.

A population-based approach to evaluating mental health services thus addresses the limitations of focusing solely on individual pre/post-symptom reduction outcomes. The real impact of an optimal care system is on the health of the community [107]. By examining a wider range of indicators and using them as benchmarks, this approach offers a more comprehensive understanding of the overall quality of mental health care services, including access, utilization, equity, and clinical outcomes at the level of a specific catchment area. Furthermore, it enables researchers and policymakers to identify gaps in service provision, allocate resources more effectively, and ultimately improve the quality of care for individuals with mental health disorders (Fig. **4**).

Six population-based small area indicators are shown that together can be used to inform on a population-based quality aspect of care. For example, in the north-west region of the Netherlands, mental health expenditure per inhabitant is average, whilst expenditure per patient is high, treated yearly prevalence is low, and the fraction of the expenditure spent on care for severe mental illness is high. Moreover, a relatively high fraction of the care was delivered through less costly basic mental health care, and a relatively low fraction was delivered through more costly specialist mental health care. This indicates that the region has adopted a ‘responsible’ model of focus on severe mental illness (SMI).

## CONCLUSION

The current state of mental health care in the Netherlands and other high-income countries faces a range of challenges, including fragmentation, accessibility, and a narrow focus on individual symptom reduction. To address these challenges, a collective and integrated approach to mental health care that encompasses the broader social, cultural, and existential context of mental illness is necessary. GEM is a social trial that seeks to provide such an approach, with a focus on empowering patients and promoting collaboration among various healthcare providers, social care organizations, and peer-support community organizations.

GEM will be evaluated using a population-based approach that encompasses a broad range of indicators related to mental health care consumption, social predictors, and clinical outcomes. In the end, of course, the verdict of the service fit should be evaluated by citizens. This approach offers a more comprehensive understanding of the overall quality of mental health care services, including access, utilization, equity, and clinical outcomes. By examining a wider range of indicators, this approach enables researchers, policymakers, and citizens to identify gaps in service provision, allocate resources more effectively, and ultimately improve the quality of care for individuals with mental health disorders.

Additionally, the success of GEM will rely heavily on the involvement of stakeholders, including patients, mental health professionals, social care providers, municipalities, and other relevant organizations. Cocreation and collaboration between these stakeholders will be key in establishing an integrated and comprehensive approach to mental health care that is responsive to the diverse needs of the Dutch population.

Overall, GEM represents an innovative approach to mental health care in high-income countries. By focusing on collaboration, integration, and the broader social context of mental illness, GEM has the potential to transform the way mental health care services are provided and improve outcomes for patients. The population-based approach to evaluation will provide valuable insights into the effectiveness of the intervention and help inform future mental health policies and practices. Ultimately, the success of GEM will depend on the commitment and engagement of all stakeholders and their willingness to embrace a collective and integrated approach to mental health care.

Overall, our analysis suggests that a more holistic, collaborative, and integrated approach is needed to address the challenges facing the Dutch mental health care system. The GEM model provides a promising framework for guiding local mental health care transformation and fostering sustainable development. By engaging stakeholders from across the ecosystem and drawing on best practices from transitions and system innovations, the GEM model has the potential to promote a more collaborative, coordinated, and patient-centered approach to mental health care that takes into account the broader social and contextual factors that contribute to mental health issues.

Furthermore, the potential of digital platforms such as psychosenet.nl and proud2bme.nl to complement traditional mental health care services and enhance public mental health in the Netherlands cannot be understated. As pro-recovery-focused eCommunities, these platforms offer accessible online support for individuals experiencing psychosis or eating disorders, respectively, and provide a wealth of information, self-help tools, and opportunities to connect with peers and professionals. Moreover, they foster a sense of belonging and social support while breaking down the stigma and isolation often associated with mental health issues.

GEM focuses on the transformation of care and support in the Netherlands. As a result, the change in society that is needed falls outside the scope of this article. Future research should, therefore, also focus on the socio-cultural aspects of health and how the government contributes to a society in which human values ​​aimed at quality of life and sustainability are at least as important in policy as economic values ​​aimed at growth and profit.

Transforming the mental health care system towards a values-driven ecosystem rooted in sustainable development goals can potentially help reverse the current trend of personnel shortages and professionals leaving the finance- and efficiency-driven mental health system. By focusing on a more holistic approach that empowers patients, promotes collaboration, and utilizes naturally available resources in the community, the mental health care system can become more appealing to current and prospective personnel. Emphasizing shared values and sustainable development goals within the ecosystem fosters a sense of purpose and commitment among professionals, which can positively influence job satisfaction and retention. Additionally, by providing a more flexible, modular, and collaborative approach, professionals have the opportunity to work across the ecosystem, reducing burnout and increasing engagement. Furthermore, the integration of digital platforms and expansion of recovery colleges will enhance the system's capacity and accessibility, potentially attracting more personnel to work in the mental health care sector.

In conclusion, the Dutch mental health care system, like in many high-income countries, faces a range of challenges that contribute to fragmentation and inequities in access and quality of care. However, by embracing a social trial piloting a more holistic, collaborative, and patient-centered approach and leveraging digital platforms to enhance public mental health, the Dutch mental health care system can examine how to overcome these challenges and provide more equitable, accessible, and high-quality care to individuals.

## Figures and Tables

**Fig. (2):**
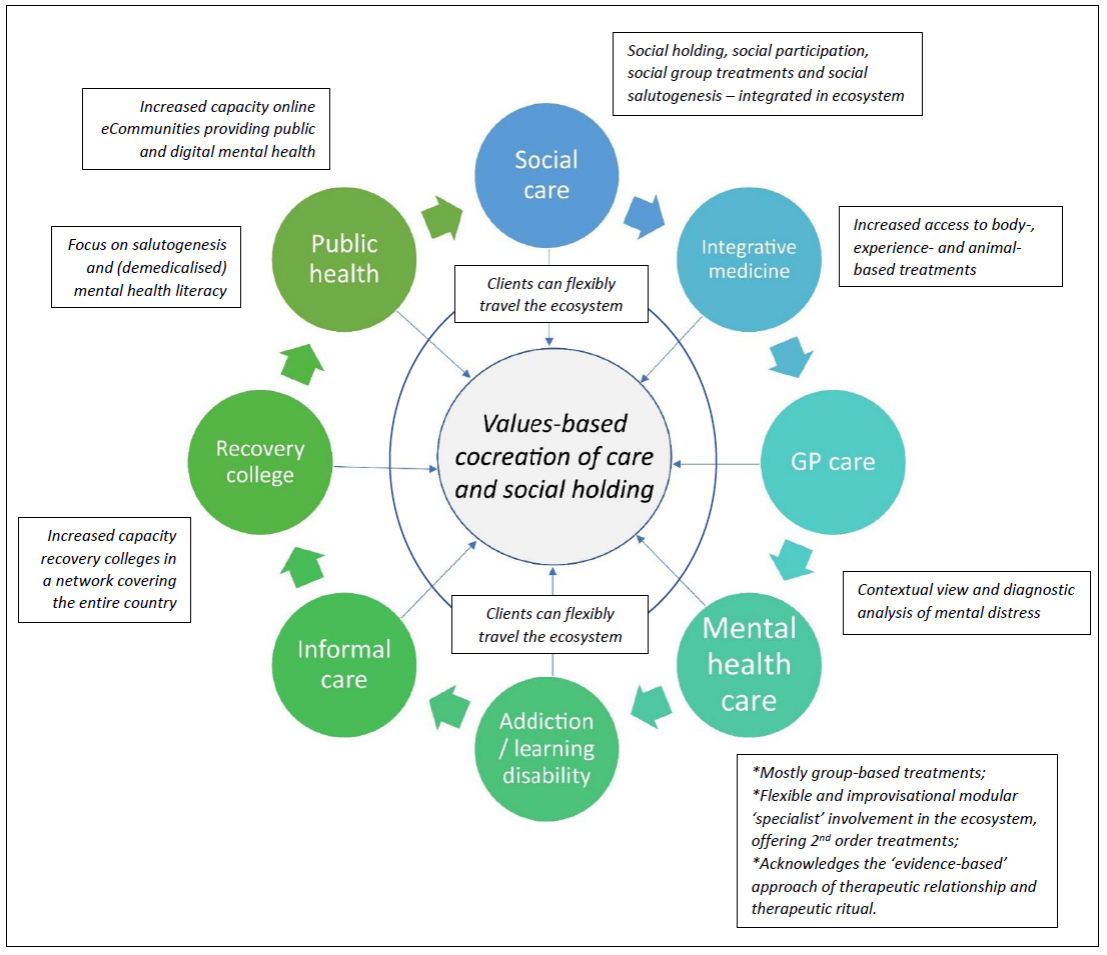
GEM model of public mental health collaboration, and main qualitative changes.

**Table 1:** Main differences between individual and collectivistic approaches towards mental health care.

**Individual Specialist Approach**	**A Collectivistic Public Health Approach**
Emphasizes specialist-based individual diagnosis and treatment	Emphasizes community-based preventive as well as group and population-level interventions
Can perpetuate power imbalances between healthcare providers and patients	Emphasizes a collaborative approach to care that empowers patients through peer-support, involvement of people with lived experience, and cocreation with users
Based on ‘evidence-based’ professional knowledge	Based on both experiential and professional knowledge
Does not reduce the overall burden of mental illness	Interventions can address risk factors and promote protective factors as they impact a wide range of people, leading to improved mental health outcomes at the population level.
Can lead to a narrow biological understanding of mental illness that is limited to individual symptoms in DSM categories	Recognizes the importance of understanding mental illness within a broader social, cultural, and existential context, with an emphasis on diagnostic cocreation with people with lived experience and promoting mental health literacy
Focuses on hospital admission, medical interventions, and therapies	Focuses on social holding, health promotion, health literacy, resilience, protective factors, non-medical early intervention, and risk reduction of social determinants of mental health (poverty, loneliness, disconnectedness, social defeat, discrimination)
Favors one-on-one treatment in the clinical setting	Favors group approaches in the community setting
The specialist determines treatment indication in the clinical setting	Patients can choose between cross-sector options on how to work on problems
May result in over-reliance on medication as a treatment option	Emphasizes a range of treatment options, including medication, psychotherapy, and non-traditional approaches such as mindfulness, meditation, body-based, experience-based, and animal-based treatments, with a focus on peer-support and involvement of people with lived experience
May neglect the importance of social support and community in recovery	Emphasizes the importance of social interventions, public campaigns, social support, and community-based interventions, such as recovery colleges, peer-supported open dialogue, retreats, and respite houses in promoting social participation, social holding, mental health and recovery
May lead to economically driven fragmented care and a lack of coordination between providers	Prioritizes a values-based coordinated approach to mental healthcare, with an emphasis on collaboration between healthcare providers, social care providers, and peer-support community organizations
Can be expensive and difficult to access for those without financial means	Aimed at providing universal access to mental healthcare
Can lead to stigmatization and labeling of individuals with mental health conditions	Aims to reduce stigma around mental health and promote a more inclusive approach to care
May reinforce individualism and a focus on personal responsibility for mental health	Emphasizes the role of social and economic factors in mental health rather than individual responsibility
The perspective of private healthcare providers	The perspective of government- and community-funded healthcare providers
Focus on individual patient billing systems and quality considerations	Focus on population-based cost system and quality considerations
